# A Role of Phase-Resetting in Coordinating Large Scale Neural Networks During Attention and Goal-Directed Behavior

**DOI:** 10.3389/fnsys.2016.00018

**Published:** 2016-03-08

**Authors:** Benjamin Voloh, Thilo Womelsdorf

**Affiliations:** Department of Biology, Centre for Vision Research, York UniversityToronto, ON, Canada

**Keywords:** oscillations, phase reset, cross frequency coupling, coding, inter-areal coordination, theta, alpha, gamma

## Abstract

Short periods of oscillatory activation are ubiquitous signatures of neural circuits. A broad range of studies documents not only their circuit origins, but also a fundamental role for oscillatory activity in coordinating information transfer during goal directed behavior. Recent studies suggest that resetting the phase of ongoing oscillatory activity to endogenous or exogenous cues facilitates coordinated information transfer within circuits and between distributed brain areas. Here, we review evidence that pinpoints phase resetting as a critical marker of dynamic state changes of functional networks. Phase resets: (1) set a “neural context” in terms of narrow band frequencies that uniquely characterizes the activated circuits; (2) impose coherent low frequency phases to which high frequency activations can synchronize, identifiable as cross-frequency correlations across large anatomical distances; (3) are critical for neural coding models that depend on phase, increasing the informational content of neural representations; and (4) likely originate from the dynamics of canonical E-I circuits that are anatomically ubiquitous. These multiple signatures of phase resets are directly linked to enhanced information transfer and behavioral success. We survey how phase resets re-organize oscillations in diverse task contexts, including sensory perception, attentional stimulus selection, cross-modal integration, Pavlovian conditioning, and spatial navigation. The evidence we consider suggests that phase-resets can drive changes in neural excitability, ensemble organization, functional networks, and ultimately, overt behavior.

## Introduction

The brain’s response to the external world is determined by its ongoing and continuously changing functional connectivity (Kopell et al., 2014). This functional connectivity often becomes evident in oscillatory activity fluctuations that are coordinated at microscopic as well as at macroscopic levels. Such rhythmically synchronized activity in the brain is widely believed to be instrumental in the formation of transient coalitions of neurons that guide behavior (Buzsáki, 2010; Fries, 2015; Womelsdorf and Everling, 2015). A key assumption of this belief is that neural coordination is controlled through intrinsic circuit mechanisms in decentralized ways (Fries, 2005; Womelsdorf et al., 2014b); although external cues affect internal dynamics, brain-intrinsic processes define the start and end of processes that implement goal directed functions such as attention, spatial navigation, or memory (Womelsdorf and Everling, 2015). One possibility is that oscillatory phase resets are such brain-intrinsic processes. Phase resetting is the re-alignment of phases of an ongoing oscillation in relation to a particular (endogenously or exogenously generated) reference point. It is evident in rodents (Courtin et al., 2014), primates (Jutras et al., 2013; Voloh et al., 2015), and humans (Rizzuto et al., 2003; Rutishauser et al., 2010), during a variety of task contexts including attention (Voloh et al., 2015), Pavlovian conditioning (Courtin et al., 2014), perceptual detection (Dugué et al., 2011), exploration (Hoffman et al., 2013; Jutras et al., 2013), learning (Rutishauser et al., 2010), speech detection (Gross et al., 2013) and memory formation (Rizzuto et al., 2003; Jutras et al., 2013). Visual and auditory stimuli predictive of rewards can both drive frequency-specific phase resets in their respective cortices (Kayser et al., 2008; Lakatos et al., 2008; Schroeder and Lakatos, 2009), as well as the anterior cingulate cortex (Voloh et al., 2015), and the hippocampus (Mormann et al., 2005). Endogenous signals representing exploratory saccades (Hoffman et al., 2013; Jutras et al., 2013), fixations (Rajkai et al., 2008), and erroneous expectations of rewards (Hyman et al., 2011) can also serve as “internal cues” that can reset phase. In recent year, many notable reviews on oscillatory coordination have detailed its neurophysiological underpinnings (Buzsáki, 2006; Wang, 2010; Lisman and Jensen, 2013; Womelsdorf et al., 2014b), implications for coding (Panzeri et al., 2010; Akam and Kullmann, 2014; Fries, 2015), emergence when attention, decision making and choice demands increase (Womelsdorf et al., 2010b; Siegel et al., 2012; Gregoriou et al., 2015; Watrous et al., 2015b), as well as its clinical relevance (Thut et al., 2011; Voytek and Knight, 2015). Here, we seek to extend these valuable contributions by surveying recently gathered evidence in light of dynamic resets, or realignments, of the phases of periodic activity fluctuations among neural circuits at the very moment when these circuits implement specific attention and memory functions.

In particular, this review seeks to highlight four aspects. Firstly, we outline how oscillations are often correlated with known anatomical and functional patterns of connectivity, suggesting that tracking dynamic changes in oscillations can serve as a proxy for changes in connectivity. Secondly, we survey how aligning oscillation phases to salient cues enhances the encoding and transmission of behaviorally relevant information. Thirdly, we highlight evidence showing how canonical circuit elements can serve as the causal source of the correlational findings underlying phase aligned neural coordination. Finally, we discuss methodological strengths and weakness in analyzing phase resets and cross frequency interactions following from realignment of low frequency phase modulations. In the following, we survey particularly those studies that provide bridges between these three levels.

## Oscillations Can Reflect the Coordination of Specific Laminar Circuits

The global coordination of local processes that support various cognitive processes often correlates with the a rhythmic mode of operation (van Atteveldt et al., 2014). In this section, we will introduce correlational evidence of frequency specific interactions within and between neocortical lamina. This framework will be the basis in later sections on how phase resetting can be a marker of encoding and information transfer (see “Phase aligned activation supports long-range encoding of information” Section), and how perturbation of canonical circuits elements may lead to predictable functional connectivity changes (see “Motifs and computational models of phase-dependent coordination” Section).

**Figure 1:**
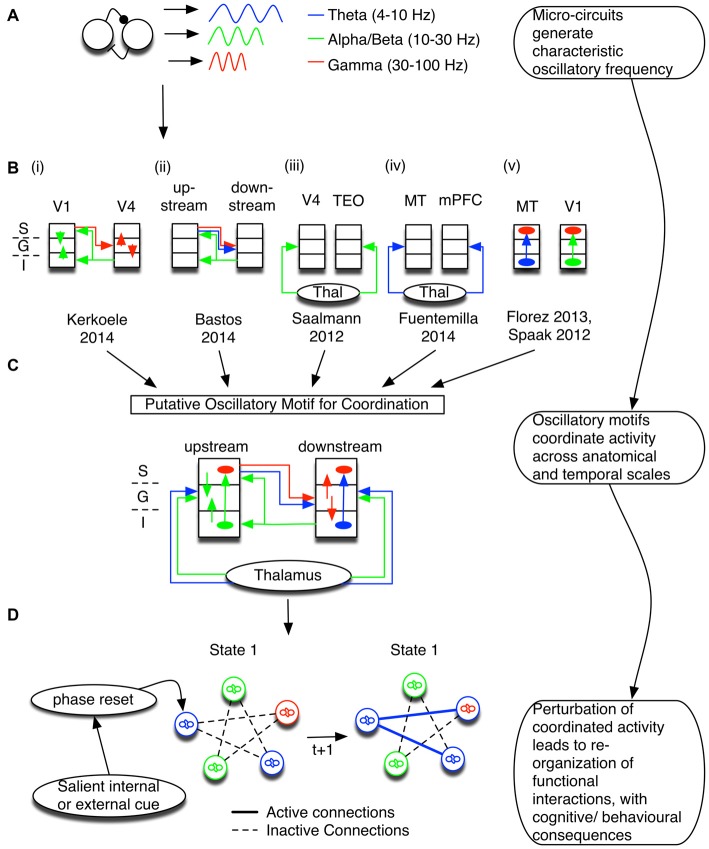
**Coordination of information flow is maintained by oscillatory dynamic circuit motifs. (A)** Canonical circuit motifs give rise to frequency-specific oscillations, such as theta (blue), alpha and/or beta (green), and gamma (red). **(B)** These motifs are embedded within cortical micro-columns and support information processing between layers and cortical areas. Summarized here are the results of six studies that suggest specific relationships of oscillatory activation signature and anatomical circuit structure. The left most inset depicts the cortical layers S, G, and I, corresponding to the supra-granular, granular, and infragranular layers, respectively. **(i,ii)** In visual cortex, gamma activity follows feedforward connections (red; van Kerkoerle et al., 2014; Bastos et al., 2015) and co-occurs with low frequency theta (Bastos et al., 2015). On the other hand, alpha/low beta activity correlates with the feedback direction (green; Bastos et al., 2015). **(iii,iv)** The entrainment of cortical oscillations may depend on thalamic input at theta, alpha and/or beta band frequencies (Saalmann et al., 2012; Fuentemilla et al., 2014). **(v)** Long-distance oscillatory coordination can then affect processing within a micro-column; for example, theta activity and alpha generated in deep layers modulated gamma activity in superficial layers (Spaak et al., 2012; Florez et al., 2013). **(C)** The six studies outlined in **(B)** have been combined to highlight a putative oscillatory motif coordinating distant sites via interactions between different frequencies. The putative combined motif suggests that a mixture of oscillatory dynamic circuit motifs coordinates information processing between cortical laminae and across brain areas. Different circuit motifs are responsible for generating/propagating specific oscillations, and are recruited to fulfill specific functions. Note that this conceptual model is dominated by studies of visual processing. We stress that the purpose of combining these studies is to set out a framework to understand how local and distant circuits may functionally interact. Thus, it is likely that brain areas with different laminar structures have a different coordination profile, and future studies across multiple brain areas are necessary to test the predictions of such a framework (see Box 1). **(D)** Not only are oscillations constrained by anatomy, but oscillatory networks also emerge in relation to specific task contexts. Tracking changes in the phase of interacting oscillatory sources can thus be used to make predictions about anatomical and neurophysiological mechanisms underlying functional changes. Abbrevations: TEO, temporo-occipital; MT, medial temporal; mPFC, medial prefrontal cortex.

Frequency-specific activity emerges from circuit interactions that can often be localized to specific cortical layers of the neocortex (Wang, 2010; Womelsdorf et al., 2014b). This activity typically falls within the delta (1–4 Hz), theta (4–8 Hz), alpha (8–16 Hz), beta (16–30 Hz), and gamma (30–100 Hz) range (Figure 1A). Slower oscillations (particularly theta, alpha, and beta) are prevalent in infragranular layers (5, 6), whereas faster gamma oscillations have been reported consistently in granular layer 4 and supragranular layers 1 and 2/3 (Bollimunta et al., 2008; Wang, 2010; Buffalo et al., 2011; van Kerkoerle et al., 2014; Ninomiya et al., 2015). Such a segregation of oscillation frequency between layers could relate to the different connectivity types across layers. Consistent with this suggestion, early analyses defined feedforward type projections as originating predominantly from the superficial layers, while feedback type projections originated in deep layers (Felleman and Van Essen, 1991). Accordingly, frequency-specific oscillations could be signatures of laminar specific anatomical projections between micro-columns in the neocortex (Wang, 2010; Buffalo et al., 2011; Bastos et al., 2015).

Rhythmic activation of these lamina-specific circuit motifs are implicated to index the flexible routing of information in support of myriad cognitive functions that depend on long-range coordination of local specialized processes (Fries, 2009; van Atteveldt et al., 2014). As outlined in Figure 1B, various studies point to oscillatory activity as a marker of layer-specific input-output coordination. At early visual cortical processing stages of macaques, gamma oscillations were evident in layer 4, the main target of feedforward connections, and spread to superficial and deep layers (van Kerkoerle et al., 2014; see also Buffalo et al., 2011). Slower alpha oscillations, on the other hand, emerged in layers 1/2 and 5, the main target of feedback connections in visual cortex, and tended to spread to granular layers. The spreading activity of gamma oscillations proceeded on feedforward connections, whereas alpha activity spread strongest on feedback projections (Figure 1Bi; van Kerkoerle et al., 2014). Varying task-demands changed the strength of activation, but not the pattern of functional connectivity, suggesting that oscillatory activity propagated along conserved anatomical constraints. Importantly, the authors showed a Granger-causal influence of feedforward/feedback projections between V1 and V4. The frequency-specific coordination in the gamma and alpha range along the visual hierarchy has been extended to higher order areas in parietal cortex and including prefrontal area 8A (Figure 1Bii; Bastos et al., 2015). Thus, the specific activation frequency of observable oscillations across the visual processing hierarchy is linked to the underlying anatomical connectivity in the visual systems (for a consideration of connectivity schemas in other brain systems see Box 1).

Box 1 **Connectivity Schemas in Other Brain Areas.**

It is an important question for future research to test whether the connectivity type between brain areas also predicts the frequency profiles of oscillatory interactions across higher association cortices that are not hierarchically structured as the visual cortical pathways (e.g., Godlove et al., 2014; Barbas, 2015). A recent, general “structural model for [anatomical] connections” proposes that the input/output pattern of feedforward/feedback projections closely depend on the divergence of laminar type, possibly independent from the hierarchical relation of the connected areas (Barbas, 2015). This model predicts that feedback-type projections originate in areas with a less elaborate laminar structure (defined by the number and distinctiveness of layers) and terminate in areas with a more elaborate structure, whereas the reverse is true for feedforward-type projections. The model can also account for the specificity of distant connections (for example, strong connections from occipital visual areas to area 8, but not to area 46).

In addition to feedforward- and feedback-type connectivity, functional cortico-cortico connections are also realized by cortico-thalamic loops that support oscillatory coordination, particularly in the theta and alpha bands (Figures 1Biii,iv; Saalmann et al., 2012; Fuentemilla et al., 2014; Malekmohammadi et al., 2015). Thalamic inputs can entrain upstream and downstream areas, facilitating the transmission of information by aligning periods of distant areas such that incoming/outgoing information can be optimally processed. Interestingly, in the supplementary eye fields (SEF)—an area with weak thalamic inputs and thus a smaller/nonexistent granular layer—the observation that infragranular slow oscillations are typically co-active with supragranular high frequency oscillations does not hold. Instead, slow and fast oscillations interact in layer 3, which is thought to follow from the SEF’s propensity for input and output connections to go through the same lamina (Ninomiya et al., 2015). Although detailed knowledge of specific laminar connectivity remains an active area of research, we would expect to observe specific frequencies to index laminar specific activation between interconnected areas.

While frequency specific activity is linked to the laminar and anatomical projections *between* micro-columns, evidence also shows that oscillations *within* a column interact (Figure 1Bv; Bollimunta et al., 2008; Spaak et al., 2012; Florez et al., 2013; Lee et al., 2013; McGinn and Valiante, 2014; Ninomiya et al., 2015). For example, in a study of human temporal neocortical slice, high frequency gamma activity was present in superficial layers. On the other hand, low frequency theta activity was evident in both supra- and infragranular layers. The phase in the former lagged behind the phase in the latter, suggesting a directional influence of theta from deep to superficial layers (Florez et al., 2013). Importantly, gamma activity was strongly modulated by the phase of theta (Florez et al., 2013; McGinn and Valiante, 2014). These results convincingly demonstrate in human cortical slices that superficial layer high frequency activity is modulated by deep layer low frequency sources.

Long-range oscillatory coordination between distant cortical columns may also affect oscillatory coordination within local columns (Figure 1C). Correlational evidence supporting such an interpretation is the nesting of high frequency amplitude fluctuation in local cortical columns within a slow frequency phase between distant sources (termed “phase amplitude, cross frequency coupling”, see “Phase aligned activation supports long-range encoding of information” Section below; Sirota et al., 2008; Tort et al., 2009; Bosman et al., 2012; van der Meij et al., 2012; López-Azcárate et al., 2013; von Nicolai et al., 2014; Voloh et al., 2015; Voytek et al., 2015). High frequency activity pulsed to the specific low frequency phase at distant sources may depend on widespread phase coherence of the slow oscillation (von Nicolai et al., 2014; Voloh et al., 2015), although phase coherence (between distant sites) on it’s own may not be sufficient to predict behavior (Voloh et al., 2015). Interestingly, feedforward-mediated information propagation at slower theta oscillation frequency has been reported to be accompanied by gamma frequency coherence across multiple visual processing stages (Bastos et al., 2015). Such a cross-frequency correlation of oscillatory activity at different frequencies has been implicated in a variety of different functions, including spatial navigation (Lisman and Jensen, 2013), attention switching (Voloh et al., 2015), learning (Tort et al., 2009), and working memory (Axmacher et al., 2010). These functional studies suggest that oscillations index both the coordination of local activity, and the dissemination of activity across large distances (Fries, 2009).

According to this interpretation, tracking such coordination, particularly in response to the onset of salient stimuli, is directly informative about internal state changes of the interacting circuits, as illustrated in Figure 1D. Perturbation of oscillatory generators may facilitate state changes in the overlying network. Aligning the phase of ongoing oscillations relative to salient cues leads to the reorganization of interacting circuit motifs between distant nodes, which may manifest themselves as coherence in the same frequency, and/or as cross-frequency interactions between frequencies. Thus, under the hypothesis that band-limited oscillatory circuits show laminar specificity (Figures 1A–C), tracking changes in the phase can be used to infer the causal source of observed changes in the functional interactions that underlie cognition and behavior. In the following sections, we outline in detail how phase resetting: (1) could aid in coding and transmitting information between distant nodes; and (2) depends on specific micro-circuit interactions.

## Phase Aligned Activation Supports Long-Range Encoding of information

Coordination across circuits can be realized by (re-) aligning or (re-) setting the phase of oscillations to an endogenous or exogenous reference point (Fries, 2005; van Atteveldt et al., 2014). Doing so permits: (1) the proper readout of stimuli encoded in the phase; (2) the transmission of multiplexed information over large anatomical distances; and (3) nesting of high frequency activity in low frequency phase that increases the informational content of neural signals.

**Figure 2:**
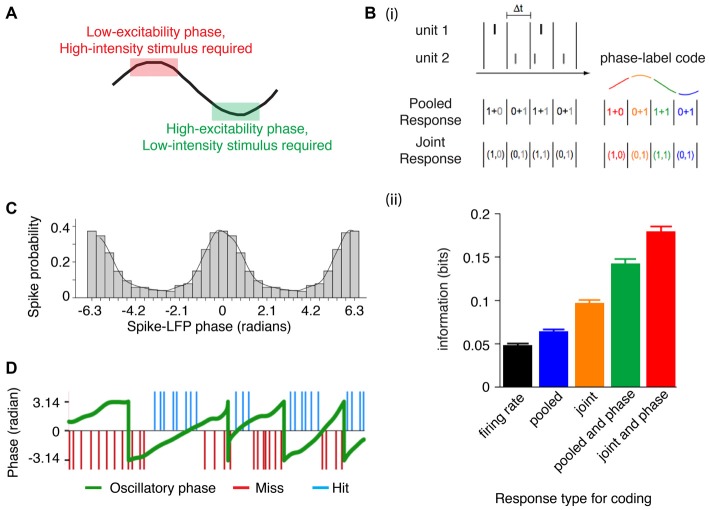
**Phase alignment permits integration of information. (A)** Stimuli can be encoded even before the phase of maximum excitation (i.e., if a strong depolarization occurs when the local circuit is resistant to perturbation), whereas stimuli with a weaker activation are encoded closer to the high excitability phase (when the circuit is sensitive to perturbations). **(Bi)** Neural codes that rely on temporal precision either pool the response of spatially distributed neurons (pooled response code) or maintain the spatial distribution responses (joint response code; left). However, taking the phase of a population oscillation can be leveraged to provide another dimension to coding (right). **(ii)** In the auditory cortex of macaques listening to natural sounds, accounting for the phase using either a pooled or joint response increases the informational content of the neural representation (illustration adapted from Kayser et al., 2009). **(C)** Phase-dependent rate coding is evident in area V1, where the firing rate increases associated with a preferred orientation occur during a specific phase of the gamma cycle. In other words, orientation selectivity is dependent on the phase of ongoing gamma (adapted from Womelsdorf et al., 2012). **(D)** Near threshold somatosensory stimuli are detected when the stimulus occurs during the rising phase (but not the falling phase) of an ongoing infra-slow oscillation (~0.1 Hz). This further suggests that encoding of somatosensory stimuli depends on the phase of the oscillation (figure adapted from Monto et al., 2008).

By aligning the phase of separate oscillators, pre-synaptic potentials from one will arrive during a time when they can have the maximal impact on a post-synaptic neuron (Fries, 2005; Womelsdorf et al., 2007). Aligning the phase thereby results in predictable windows for integration, and provides a way for an upstream reader to segregate information that should be processed from that which should be ignored simply by shifting the relative phase (Fries, 2009). For example, during a low excitability phase (when local circuitry is less sensitive to perturbation), relatively stronger incoming excitation is necessary to elicit a post-synaptic spike, whereas relatively weaker excitation is sufficient during the period of high excitability (Vinck et al., 2010). Thus, a low (high) intensity stimulus would be encoded in the high (low) excitability phase (Figure 2A). Put another way, information contained in local circuitry can be read out by a distant downstream reader if it has the same “neural syntax” as the local sender (Buzsáki, 2010), where the syntax in this case is predicated on precise phase relations between sender and reader (Maris et al., 2013).

The consequences of phase specific sensitivity to input is that information can be encoded as a function of the phase of oscillating neuronal populations. Accordingly, phase-coding or phase-of-firing coding has been shown to increase the informational content of spikes (Figure 2B; Montemurro et al., 2008; Kayser et al., 2009), and to carry information about stimulus orientation (Figure 2C; Vinck et al., 2010; Womelsdorf et al., 2012), objects in memory (Siegel et al., 2009), object and spatial navigation parameters (Huxter et al., 2008; Turesson et al., 2012). Phase resetting could thus realign fluctuating periods of excitability to match informational content about goal relevant stimuli. This also implies that stimulus encoding can be suppressed if aligned to an improper phase, as has been demonstrated for gamma frequency specific gating of stimuli between V1 and V4 during selective attention (Bosman et al., 2012). Such a gating function of phase synchronization is not restricted to gamma frequencies. For example, studies have shown improved perceptual stimulus detection as a function of the oscillatory phase of infra-slow frequency activity (Figure 2D; Monto et al., 2008), as well as theta and alpha frequency activity (Palva et al., 2005; Busch et al., 2009; Bonnefond and Jensen, 2012, 2013) during stimulus presentation and that saccadic reaction times to a near-threshold stimulus decrease when the stimulus is presented at an optimal theta phase (Diederich et al., 2014). Successful reactivation of remembered words (presented on a background flickering at either theta or alpha periodicity) can be predicted from the phase of neural activity at the same frequency (Wimber et al., 2012). Externally controlling the onset of alpha and theta oscillations via transcranial entrainment has similar effects on perceptual detection (Dugué et al., 2011) and value based decision making (Polanía et al., 2012, 2015). Importantly, internal oscillations governing stimulus detection are not just passive processes but can be internally controlled. For example, if two stimuli are presented, one may be preferentially encoded if an oscillation has been reset such that an optimal phase matches one but not the other. (Lakatos et al., 2009; Schroeder and Lakatos, 2009; Landau et al., 2015; see “Cross-modal attentional integration and selection” Section). Taken together, phase realignment has been directly linked to the selection of stimuli and the encoding of stimulus information.

Going one step further from the selection of single stimuli, phase-aligned oscillatory activity also permits multiplexing, i.e., the encoding and decoding of multiple information streams at the same time, either via a frequency or time division of oscillations (Akam and Kullmann, 2014). In *frequency division multiplexing*, activity in separate frequency bands carries separate information. A downstream reader could then parse information by matching the frequency of the relevant oscillation (Akam and Kullmann, 2010). A signal can be decoded by evaluating shifts in the phase of the input relative to a reference oscillation (Panzeri et al., 2010; although other forms of frequency division multiplexing can also evaluate shifts in the *amplitude* of the incoming signal, see Akam and Kullmann, 2010). In this scheme, phase resetting of circuit motifs responsible for instantiating the reference oscillation is critical for the proper readout of an incoming signal. Studies in hippocampus and cortex indeed suggest that different oscillations are correlated with different information, suggesting that frequency-specific oscillations themselves may reflect segregation of neuronal processes encoding separable information (Colgin et al., 2009; Buschman et al., 2012; O’Connell et al., 2014; Womelsdorf et al., 2014b). A reference oscillation may be instantiated via circuits that resonate at intrinsic frequencies (Panzeri et al., 2010); for example, layer 5 pyramidal cells can generate theta activity independent of external inputs (Silva et al., 1991; Florez et al., 2013). A complementary mechanism may independently reset oscillations of different frequencies in sensory cortices (O’Connell et al., 2014), perhaps through the selective targeting of distinct microcircuits with resonance or pacemaking preference for different frequencies. In other words, aligning the phases of separate frequency-specific oscillations may provide a gating mechanism that permits multiple streams of activity to propagate simultaneously in the same cortical column (Womelsdorf et al., 2014b). This conclusion is consistent with a recent proposal suggesting that the alternate recruitment of cortico-thalamic networks may depend on different oscillations to fulfill specific functions (Ketz et al., 2015).

**Figure 3:**
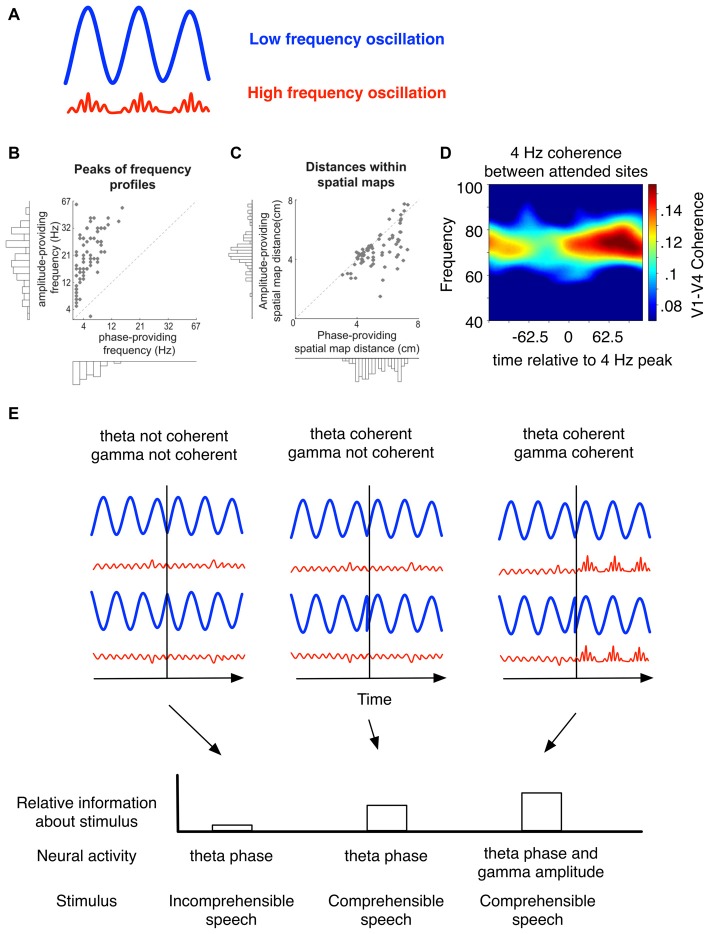
**Nested oscillatory interactions route local and long-range activity to optimize stimulus representation. (A)** The phase of slower (e.g., theta) oscillations affect processing facilitated by faster oscillations. Theta phase can organize gamma bursts, evident as a modulation of gamma amplitude. **(B,C)** Each data point is obtained by (1) extracting unique CFC patterns based on anatomical location and frequency content; and (2) from these patterns of location and frequency content, selecting LFPs with statistically significant coupling (for details, see van der Meij et al., 2012). **(B)** The frequency peak was calculated from each unique CFC pattern as the peak of the spectral profile of coupled phase or amplitude-providing LFPs. Phase providing LFPs had a spectral peak that was characteristically lower than amplitude providing LFPs. **(C)** In the spatial map, the mean Euclidian distance between LFPs is generally greater for phase providing rather than amplitude providing LFPs. This implies that during cross frequency coordination, low frequency activity has an influence over a greater space than high frequency activity. **(D)** In macaques, coherence in the gamma band between V1–V4 increased for attended but not unattended stimuli (Bosman et al., 2012). Gamma coherence between distant sites was modulated as a function of theta phase (adapted from Bosman et al., 2012). **(E)** (Top panel) In this schematic, phase resetting in low or high frequency sources (or both) can facilitate stimulus encoding. (Bottom panel) While low frequency oscillations may emerge to support a specific function, high-frequency activity can significantly contribute to internal stimulus representation. For example, during comprehensible but not incomprehensible speech, theta phase is informative of the speech envelope. However, theta-modulated gamma amplitude significantly reduces uncertainty about the stimulus (see Gross et al., 2013). Phase-modulated gamma activity also contributes to attention switching in macaques (Voloh et al., 2015).

In contrast to frequency division multiplexing, time-division multiplexing depends on ordering packets of information (“items”) at different times relative to each other, where the temporal reference frame is determined externally (Akam and Kullmann, 2014). In neural networks, this reference frame can be internally generated by oscillatory activity aligned to a salient stimulus, i.e., by a phase reset. Accordingly, oscillations would provide a scaffold in which sequential information can be ordered according to the relative phase of a background oscillation (Panzeri et al., 2010). Oscillations with different periods could thereby organize information that is represented in the brain on multiple time scales (Panzeri et al., 2010; Ratté et al., 2013) by co-modulating their activity. Such co-modulation is often evident as cross-frequency coupling, which has been suggested to play a role in coding multiplexed signals (Lisman and Jensen, 2013; Akam and Kullmann, 2014). One particular manifestation of CFC that has received widespread attention is phase-amplitude coupling, where the amplitude of a fast oscillation varies as a function of the phase of the slower oscillation (Figure 3A; see Canolty and Knight, 2010; Hyafil et al., 2015b for a review, on other forms of cross-frequency interactions). In this context, sequential information encoded in different neural ensembles is evident in the amplitude of successive fast oscillations (such as gamma), and each item is organized according to the phase of a slow oscillation (such as theta, Lisman and Jensen, 2013; although CFC may also support frequency division multiplexing, Akam and Kullmann, 2010). In other words, the phase of the slower oscillation provides a modulatory effect on computations occurring at the speed of the faster oscillation.

Empirical evidence to date suggests that the cortex can use both time division and frequency division multiplexing, as well as “mixed multiplexing” regimes (Akam and Kullmann, 2014). Time division multiplexing seems particularly suited to encoding task variables that occur sequentially, as occurs during working memory (Siegel et al., 2009; Watrous et al., 2015a) or spatial navigation (Pastalkova et al., 2008; Mizuseki et al., 2009; Wang et al., 2015) tasks, whereby stimuli are encountered in an ordered way. On the other hand, frequency division multiplexing may be more likely to occur when stimuli are not sequentially presented, for example, in a delayed match to sample task (Liebe et al., 2012). This may be evident at the neurophysiological level as *short* bursts of oscillatory activity (Akam and Kullmann, 2014). Although, to our knowledge, there is no strong evidence of frequency division multiplexing (i.e., that an incoming multiplexed signal can be decoded via the phase of separate frequencies), indirect evidence exists in that individual neurons can lock to the phase of multiple different frequencies (Colgin et al., 2009; Canolty et al., 2010). That said, and as noted by Akam and Kullmann (2014), time-division multiplexing could still be a viable way for the transmission of population activity even when the stimulus information is not explicitly sequential.

A robust coding scheme to represent information using both frequency and time division multiplexing would require a high diversity of frequencies and phases that carry information about local processes and are able to synchronize over long distances. Intriguingly, such diversity in CFC is prevalent across the neocortex of humans (Maris et al., 2011, 2016; van der Meij et al., 2012, see also Canolty et al., 2010). van der Meij et al. (2012) found such diversity in the range of frequencies of both phase and amplitude providing LFPs (Figure 3B) and diversity in the preferred phase to which high frequency bursts coupled. During CFC, phase-providing channels were more spatially distributed than amplitude providing channels (Figure 3C). Such asymmetric distribution of low and high frequency activity can facilitate preferential routing across long-distances. These findings are in line with other studies showing long-distance coupling between sources of low and high frequency bands (Sirota et al., 2008; Bosman et al., 2012; von Nicolai et al., 2014; Voloh et al., 2015; Figure 3D). This supports the notion that both the phase and frequency are manipulated to route information contained in local nodes across spatially distributed networks.

Evidence that phase resetting plays a pivotal role in facilitating encoding via cross-frequency interactions comes from a study of MEG activity in humans listening to comprehensible vs. incomprehensible speech (Gross et al., 2013). Using an information-theoretic analytical framework, this study reported that theta phase oscillations align to sudden, large amplitude transients in the speech envelope of comprehensible as opposed to incomprehensible speech, thus aligning internal oscillations to the natural theta-rhythmicity evident in speech. Although gamma oscillations did not lock to the cue, they were locked to theta phase. Importantly, theta-gamma coupling increased after speech edges, and gamma activity (organized by theta) reduced the uncertainty about the speech envelope (Figure 3E, bottom panel). This may be because oscillations of different frequencies track separable speech components occurring at different time-scales (Giraud and Poeppel, 2012). In other words, resetting theta may have led to the reorganization of gamma activity, thus facilitating encoding of natural speech (Gross et al., 2013; Hyafil et al., 2015a). These findings suggest that stimulus-aligned cross-frequency interactions are an emergent property of neural circuits that can carry information somewhat independently of their constituent oscillations (Figure 3E, bottom panel). In line with such an interpretation, Voloh et al. (2015) showed that inter-areal theta-gamma coordination predicted correct stimulus selection vs. errors of stimulus selection, while long-distance theta coherence could not. Thus, at least in the realm of speech processing and attention, phase resetting to salient environmental cues emerges at the time when high-frequency activity modulated by low-frequency phase contributes significantly to stimulus representation.

The coding schemes outlined above are supported by studies showing slow frequency activation is often causally responsible for organizing both gamma amplitude variation and single unit activity according to the phase of the slower frequency (Pastoll et al., 2013; Pernía-Andrade and Jonas, 2014). For example, inducing optogenetic depolarization with theta pulses in rodent medial entorhinal cortex slices leads to gamma oscillations that align with the peak of theta over multiple cycles (Pastoll et al., 2013). Pharmacological inactivation of the medial septum, a major source of theta drive in rodents, destroys the ability of rats to form place fields in novel, but not previously experienced environments, suggesting that theta is critical for the generation and organization of internally generated spatial sequences (Wang et al., 2015). Importantly, during spatial navigation, spikes in the hippocampus and entorhinal cortex lock to preferred theta phases and to concurrent gamma activity (e.g., Colgin et al., 2009). Thus, it is likely that theta-mediated gamma activity is necessary to organize internally generated sequences for successful goal completion (Lisman and Jensen, 2013; Pezzulo et al., 2014).

In summary, informational content in neuronal activity is increased when the phase of oscillations is taken into account, suggesting that phase resetting oscillations serves as a in encoding goal relevant information. Nesting of faster oscillations within slower ones permits multiplexed codes that may be robust across long distances, particularly when low frequency phase is aligned to salient events. Such interactions arise via the causal interaction of distinct circuit elements necessary for the maintenance of internally generated sequences. Based on the ubiquitous evidence of phase-dependent coding and cross-frequency coupling outlined above, we speculate that phase reset mechanisms that facilitate cross-frequency coupling are a fundamental signature of the encoding of goal-relevant stimuli in and between higher cortical areas.

Functionally, such phase-reset mediated cross-frequency coupling could represent the formation of a distributed cell assembly (Canolty et al., 2010; Womelsdorf and Everling, 2015), or reflect selective neuronal communication between distant sites (per the study in Figure 3C, and as suggested in Figure 1D). In the following section, we will turn to modeling work that suggests how circuit elements responsible for the generation of oscillations at distinct frequencies can interact to form different dynamic coupling profiles for the coordination of activity between brain areas.

## Motifs and Computational Models of Phase-Dependent Coordination

The evidence that phase aligned activation plays a role in information processing across a broad range of tasks and in different species, suggests that there may be common local mechanisms and circuit motifs that mediate phase alignment and cross-frequency interactions (Hyafil et al., 2015b). In the following section, we will describe a general tripartite model illustrating: (1) how cross-frequency interactions can arise; and (2) the how phase resetting may mediate such interactions.

**Figure 4:**
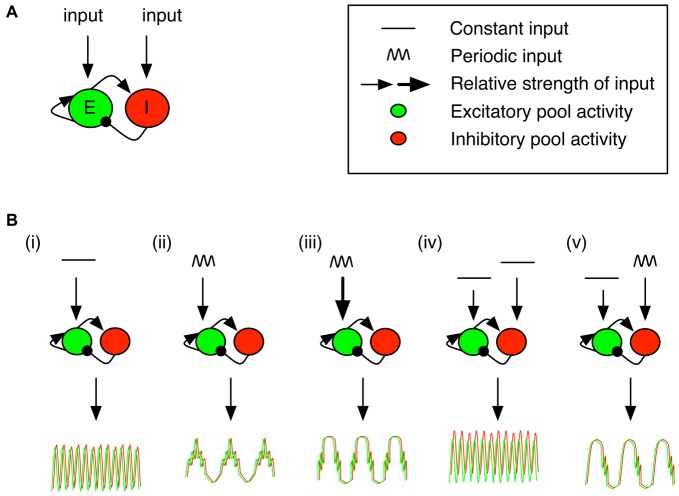
**Pyramidal-interneuron-gamma model (PING) motif with differential drives and targeted neuron populations results in differential cross frequency coupled profiles.** The PING model gives rise to cross frequency coordination (see Onslow et al., 2014). **(A)** The PING motif has been widely studied as a generator of fast gamma oscillations. Arrowheads correspond to excitatory connections, while circles correspond to inhibitory ones. The excitatory and/or inhibitory population can receive inputs. The response of the population to an input is governed by a sigmoidal response curve. **(B)** The excitatory or inhibitory populations can be targeted with a constant and/or periodic input. This panel contains a selection of response profiles, and abstracts over specific input values responsible for the observed output profile (for a full description, see Onslow et al., 2014, in particular Figures 3C, 4B,E, 5A, 6D). (From left to right) **(i)** A constant input to the excitatory population generates gamma oscillations. **(ii)** An 8 Hz periodic stimulation of the excitatory population generates a signal with the same period for pyramidal cells and interneurons, but with gamma activity locked to the peaks. **(iii)** Increasing the input past a critical point results in gamma activity locked to the ascending and descending slopes. **(iv)** Targeting both the inhibitory and excitatory populations with a constant input results in similar gamma activity as in **(i)**. **(v)** However, when the input to the inhibitory population is periodic, gamma activity locked to the descending phase of the local population.

Computational studies have shown that the pyramidal-interneuron-gamma model (PING) is a generic circuit motif that can intrinsically generate phase aligned activation through recurrent interactions of excitatory pyramidal cells and inhibitory interneurons (Onslow et al., 2014; see also Womelsdorf et al., 2014b; Figure 4A). Activation of the excitatory pool drives both excitatory and inhibitory activity. Inhibitory activity dampens excitatory activity after some delay. The frequency of the fast oscillation is determined by the strength of the excitatory drive, the (axonal) delay between excitatory firing and inhibitory activation; and the timing constant associated with fast, inhibitory GABA_A_ channels (Buzsáki and Wang, 2012). These channels are prevalent in fast spiking inhibitory interneurons, which play a critical role in biological gamma generation (Cardin et al., 2009; Sohal et al., 2009; Wulff et al., 2009).

Phase resetting of the PING circuit can arise either via tonic or phasic activation. A tonic drive to the excitatory population can be sufficient to generate population oscillations (Figure 4Bi), even in the presence of a tonic drive to the inhibitory population (Figure 4Biv; Buzsáki and Wang, 2012 see Tiesinga and Sejnowski, 2009, for an inhibition-based model). If another input provides a sufficiently strong impulse, then a phase shift would be evident in the gamma band. On the other hand, a change in the timing of a slow phasic drive (i.e., a phase reset in the *input*) can also manifest itself as a phase reset in the microcircuit, but would be detected as a phase reset in the slower frequency.

Combining phasic and tonic drive to either the excitatory or inhibitory populations can generate nesting of faster frequencies in the slower, phasic drive (Onslow et al., 2014). However, the phase at which the high frequency activity is locked to slow activity depends on the strength of the phasic drive and the activation level in the target neural population. Medium- or high-level theta activity to the excitatory population locks gamma activity to the peaks or slopes, respectively (Figures 4Bii,iii, respectively). On the other hand, phasic activity that impinges on the inhibitory population tends to force nesting of high frequency oscillations on the descending slope of the slow oscillation (Figure 4Bv). Thus, observing locking of high frequency activity to particular phases of the low frequency oscillation may allow us to infer, via these modeling results, the type of input (phasic/tonic) and the target neural population (pyramidal/fast spiking inhibitory). One intriguing possibility left unexplored, to our knowledge, is the modulation (in real time) of the phasic input. In such a case, high frequency activity would likely shift relative to the low frequency oscillation. This may be observable as a phase reset (across trials) of the high frequency activity, locked to a characteristic phase of the slow drive. Moreover, an open question is how the initial phase of oscillatory circuit activation following a reset can be modulated based on cellular targets or the strength of inputs. This would set the stage for differential processing of post-synaptic currents arriving at predictable but different times. Nevertheless, it is clear that observing phase resetting in such a generic model, combined with a specific characterization of cross-frequency interactions, can yield rich insights into the underlying circuit mechanisms.

It is important to dissociate phase resetting in the PING motif from phase resetting originating in phasic drives. In addition to the indirect evidence set out in “Oscillations can reflect the coordination of specific laminar circuits” Section, the phasic drive alluded to above (Onslow et al., 2014) is considered to originate from distinct neural components (Gloveli et al., 2005; Wulff et al., 2009; Stark et al., 2013; Fukunaga et al., 2014; Pernía-Andrade and Jonas, 2014; Hyafil et al., 2015b). To understand the causal interaction of different circuit elements responsible for phase reorganization and hierarchical nesting of oscillations, we will describe modeling work based on hippocampal circuitry. Modeling of such interactions in the cortex is limited (Lee et al., 2013; Hyafil et al., 2015a), though the intuition remains similar as for hippocampal and entorhinal circuit models; functional interactions rely on the proper coordination of low and high frequency circuit elements, which may be embedded in different lamina (see “‘Oscillations can reflect the coordination of specific laminar circuits” Section).

The models we describe are composed of a network of interconnected pyramidal cells, fast spiking interneurons (parvalbumin+), and oriens-lacunosum moleculare (OLM) cells (Tort et al., 2007; Wulff et al., 2009; Malerba and Kopell, 2013; Neymotin et al., 2013; Stark et al., 2013). These models highlight the causal interaction of different circuit elements responsible for phase reorganization and hierarchical nesting of oscillations. The first two elements compose the gamma-rhythmic PING motif we have described above, whereas OLM neurons tend to oscillate at a slower theta rhythm due to a Ca^2+^ I_h_ hyperpolarizing current, which leads to longer time constants between hyperpolarization and depolarization (Tort et al., 2007; Neymotin et al., 2013; Stark et al., 2013). Modeling work suggests that OLM cells that impinge on multiple, separate PING circuits can synchronize these local gamma rhythmic circuits (Tort et al., 2007). The phasic drive by OLM cells is critical for the segregation of gamma activity bursts by theta phase, because a strong, phasic drive by OLM cells reduces pyramidal cell activation. Thus, fast spiking inhibitory cells are not activated, and gamma is reduced during the period when OLM cells are active (Neymotin et al., 2013). In other words, the rise and fall in OLM cell activity (i.e., the phase) determines the activation of the PING circuit, with shifts in the slow frequency phase leading to differential activation of those neuronal ensembles that generate the high frequency amplitude.

What mechanism permits the modulation of pyramidal cells via phase-aligned slow frequency components? Pyramidal cell entrainment to theta rhythmic activation is likely linked to their resonance properties. Optogenetic work has shown that pyramidal neurons are broadly resonant at theta-frequencies (i.e., spiking is enhanced due to sub-threshold theta-oscillatory input; Stark et al., 2013), dependent on inhibition of fast-spiking interneurons and an additional dampening mechanism, provided, for example, by OLM cells. This suggests that phase re-alignment of the activity of neurons in a PING circuit may be restricted to intrinsic frequencies, though under the control of upstream phasic drivers. Interestingly—in hippocampal pyramidal cells—such intrinsic resonance frequency in the theta band increases with distance from the soma, and increases as a direct response to changes in synaptic plasticity (Narayanan and Johnston, 2007). Considering that projection neurons from upstream areas have preferential synaptic targets on the dendritic tree, this result suggests that individual neurons may differentially filter signals from different areas by adaptively tuning their resonance frequency (Narayanan and Johnston, 2007). Thus, even if a phasic drive is reset, it may only have an effect if the pyramidal cell population remains tuned to the same frequency.

*In vitro* and modeling work suggests that the coupling of theta and gamma via PING and OLM circuits depends on reciprocal connectivity between the two (Wulff et al., 2009; Malerba and Kopell, 2013). Although OLM cells can reset the phase of gamma oscillations, establishing a coherent theta rhythm itself depends on the feedback of fast-spiking parvalbumin+ interneurons (Wulff et al., 2009). This study also found that without coherent theta-synchronized activity, the coupling of the gamma rhythm to the theta phase was reduced. In addition to these theta/gamma interdependencies, recent modeling work suggests that when multiple PING-dependent gamma spikes occur in a theta cycle, each PING-spike modulates the theta phase (Malerba and Kopell, 2013). In this model, the theta drive subsequently resets the phase of gamma, resulting in mutually reinforced cross-frequency phase to amplitude coupling. This study underscores the dynamic nature of cross frequency coupling, particularly when all component cells of fast and slow oscillatory circuits are interconnected, as opposed to a scheme with unidirectional coupling between slow and fast oscillation generators (Hyafil et al., 2015b). It is not always the case (at least in the hippocampus) that a slower oscillation enslaves a faster one, but rather that the reciprocal connections permit the embedding of faster oscillations in slower ones, resulting in subsequent processing proceeding at a timescale determined by the slower oscillation. Such a model has been proposed for working memory maintenance, where the number of items that can be stored in working memory depends on the number of cycles of the fast oscillation that can be embedded in the slower one (Lisman and Jensen, 2013). Consistent with this, increasing the memory load also leads to a decrease in the frequency of the slow oscillation during phase-amplitude coupling (Axmacher et al., 2010). Thus, we speculate that a circuit analogous to the one we have described may more generally permit control of multi-item processing by modulating the circuitry responsible for slow frequency generation.

The surveyed theoretical studies highlight that already simple, generic circuit motifs can give rise to complex first order and second order oscillatory interactions. Importantly, these studies provide explicit predictions about the cell-type specificity of cross frequency oscillatory interactions, their sensitivity to different tonic/phasic input regimes and their dynamic unfolding over time. Only a few of these predictions have been directly tested empirically, calling upon future studies to follow explicit hypothesis driven testing of dynamic interactions of different cell types supporting faster and slower frequency synchronization during phase amplitude cross frequency coupling. We discuss in the next section the coordination of such cell specific spiking activity with population level local field potential (LFP) oscillations, and how spike to LFP interactions relate to behavior.

## Functional Implications of Phase Aligned Activity

The previous section described motifs and mechanisms of phase aligned neuronal activation, but left open how these mechanisms are recruited during goal directed behavior. The following surveys studies that illustrate direct links between periods of phase realignment and behavioral function, where (1) environmental context leads to a cue-aligned phase reset; (2) local activity is modulated by larger-scale phase reset; and (3) phase rearrangement of network dynamics lead to changed function. In the first section, we will describe functions that are recruited during behavioral adaptation, notably cross-modal attentional selection, visual attention control, learning of Pavlovian cues during classical conditioning, and spatial navigation. In a second section, we will describe how phase aligned neural activation can lead to long-term changes in synaptic plasticity.

### Behavioral Adaptation

#### Cross-Modal Attentional Integration and Selection

A host of studies has shown that internally generated oscillations can be entrained to externally imposed periodicity imposed by multiple modalities (Lakatos et al., 2009, 2013; Diederich et al., 2014; for review, see Schroeder and Lakatos, 2009; Calderone et al., 2014). The internal oscillation can be reorganized such that the upcoming stimulus is presented during the phase of high cortical excitability. For example, Lakatos et al. (2013) showed that neural ensembles in auditory and visual cortex take advantage of both the timing and frequency of the attended stream to heighten sensitivity to an oddball stimulus present in an attended auditory stream. Delta phase was opposite in neural ensembles that were tuned vs. not-tuned to the frequency of the attended stream. Importantly, in attended sensory streams, delta phase was facilitative in neural ensembles tuned to the frequency of the attended stream, but inhibitory in ensembles not tuned to the stream. Reorganization of delta phase was suggested to originate from phase resetting, because delta power did not change during rhythmic input, and phases were aligned (across trials) for a longer period then the stimulus presentation. These results illustrate that low frequency phase resets in primary auditory and visual cortices are under endogenous attentional control (Lakatos et al., 2009). Moreover, cross-modal interactions may be subserved by a phase reset that is mediated by the dominant area (e.g., when the stimulus is visual, V1 exerts an influence on A1 by phase resetting oscillations in A1). As Lakatos et al. (2013) hypothesize, a phase reset in A1 is likely mediated by oscillations originating in the thalamus.

#### Attentional Stimulus Selection

As mentioned in previous sections, studies of rhythmic entrainment in sensory cortices suggest that endogenous “top-down” attentional signals can instantiate a phase reset (Lakatos et al., 2009, 2013). Visual cortices can be rhythmically entrained to frontal cortices (Gregoriou et al., 2009; Liebe et al., 2012; Bastos et al., 2015), with oscillations in the frontal cortex likely emerging first and visual cortex lagging behind (but see Salazar et al., 2012). Moreover, frontal oscillations may organize oscillations in sensory cortices during endogenous attentional control such that neurons fire more coherently (Gregoriou et al., 2009). In fact, oscillations emerging in the frontal cortex of human or nonhuman primates are implicated to support interregional communication across various task demands such as perception (Gross et al., 2004; Hipp et al., 2011; Micheli et al., 2015), working memory (Liebe et al., 2012; Salazar et al., 2012), response inhibition control (Phillips et al., 2014), rule switching (Womelsdorf et al., 2010a; Buschman et al., 2012), attentional selection (Siegel et al., 2008; Voloh et al., 2015), and decision making (Pesaran et al., 2008). A distinction between these studies and studies of encoding in sensory processing and speech cognition is that the stimulus does not have to be rhythmic for functional oscillations to emerge. Instead, such oscillations emerge self-generated, possibly supporting the organization of internally generated sequences (Pezzulo et al., 2014).

**Figure 5:**
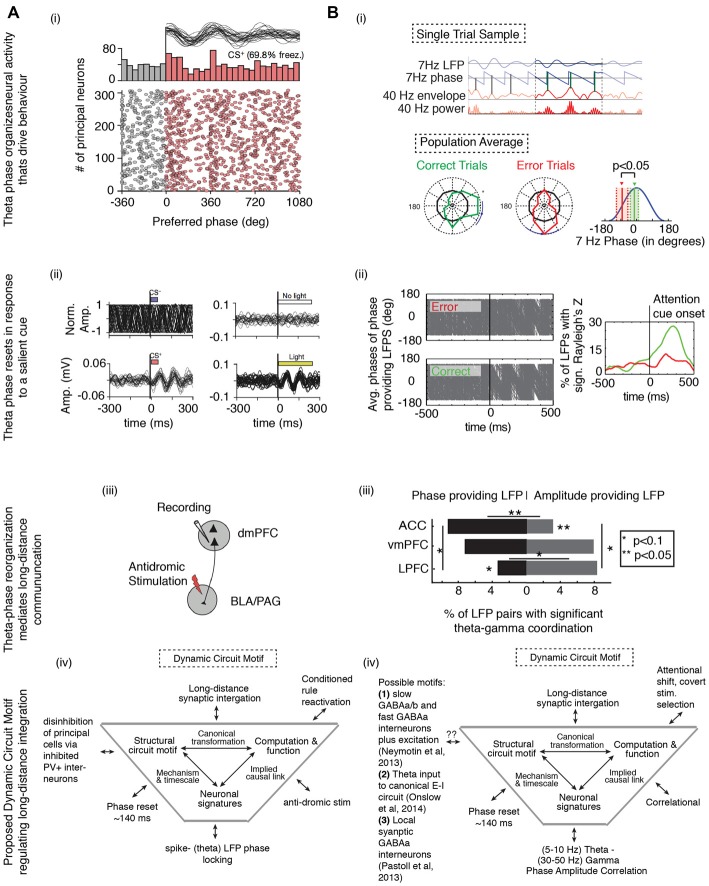
**Theta phase alignment to salient cues coordinates networks and correlates with behavioral success.** Cue-aligned theta oscillations in the medial prefrontal cortices **(i)** organizes neural activity that drives behavior when **(ii)** the low frequency phases reset in response to a salient cue, and **(iii)** routes information across long-distances, all of which **(iv)** can be understood in terms of a Dynamic Circuit Motif, visualized near the bottom of the figure (for a review, see Womelsdorf et al., 2014b). **(A)** In a Pavlovian conditioning task, rodents learn to associate a neutral cue (conditioned stimulus, CS) with an aversive stimulus. Recordings were made in the rodent mPFC (adapted from Courtin et al., 2014). **(i)** Theta oscillations were often apparent when the animals exhibited freezing behavior. Neural spikes were locked to theta peaks, suggesting that when present, theta oscillations drove local circuit modulation that resulted in a fear response. **(ii)** Cue-aligned theta oscillations emerged in response to cue onset (left), but only when the animal was in a high (bottom) but not low (top) fear state. Optogenetically inducing theta oscillations also led to freezing (right). **(iii)** Neurons reorganized by theta projected to targets responsible for instantiating the fear response. Thus, theta phase reset in upstream areas is critical for affecting fear processing in downstream areas. **(iv)** A proposed dynamic circuit motif relating the reactivation of a conditioned rule (the function) to spike-theta phase locking (the neural signature). The long distance synaptic integration is achieved by inhibiting PV+ interneurons, leading to the disinhibition of projecting excitatory principal neurons. **(B)** In a cued selective attention task, macaques had to covertly shift the focus of attention to a target in order to correctly identify a change in the target (adapted from Voloh et al., 2015). **(i)** Following onset of the cue that triggered the attention shift, high frequency gamma amplitude was locked to the peaks of theta oscillations in prefrontal and anterior cingulate cortex. Importantly, gamma-amplitude to theta phase locking on correct trials was systemically different than on error trials. **(ii)** Correct, but not erroneous, covert attention shifts were also accompanied by an increase in theta phase consistency in those LFPs that modulated gamma activity. **(iii)** During theta-gamma correlation, LFPs with theta information predominated in the ACC, while those with gamma information were more likely in the LPFC. This study suggests that theta modulation of local activity may extend to distant sites. In line with the study in **(A)**, theta phase reset drives neural reorganization, leading to observable behavioral changes. **(iv)** In the framework of a dynamic circuit motif, where a function (attention switching) is correlated with a neural signature (theta-gamma correlation). Causal connection between them could be subserved by neural elements known to generate gamma nested in theta oscillations. Abbreviations: BLA, basolateral amygdala; PGA, periaqueductal gray area; PV+, parvalbumin+.

Endogenous control of dynamic neuronal coordination between distant structures via phase resetting has recently been documented in the macaque during a selective attention task (Voloh et al., 2015; Figure 5B). This study examined how internal rhythmic dynamics support attentional deployment in anterior cingulate and lateral prefrontal cortices. Theta activity aligned between anterior cingulate cortex and lateral prefrontal cortex at the time of covert stimulus selection, and the successful deployment of an attention network was indexed by the correlation of theta phase with gamma amplitude. Importantly, this coordination was evident on correct trials, but not error trials, and it emerged at the same time when neurons in prefrontal and anterior cingulate cortex show selectivity for stimulus features (Kaping et al., 2011; Figure 5Bi). This effect was likely mediated by a phase resetting mechanisms, because theta-modulating LFPs exhibited a spectral peak in the theta band that did not change before and after attention cue onset, whereas theta phase consistency increased after attention cue onset (Figure 5Bii). Theta LFPs were more likely to originate in the anterior cingulate cortex, whereas gamma LFPs were more likely to be found in the LPFC (Figure 5Biii). Moreover, the same local circuits that provided theta phases that correlated with gamma band activation after attention cue onset also hosted neurons that fired bursts of spikes that synchronized to distant gamma band activity (Womelsdorf et al., 2014a). In particular, the strength of long-range burst-LFP synchronization correlated positively with the degree of theta-gamma coordination (Voloh et al., 2015). Local bursting synchronized to distant gamma could have driven theta reorganization, or it could have been the result of reorganized theta that focused neuronal firing. In either case, similar to results from a study on cue triggered fear response in a Pavlovian paradigm in rodents (see below; Courtin et al., 2014), the theta phase reset predicted when local neuronal activity was successfully coordinated between long distant circuits (Figure 5Biv).

#### Reactivation of Learned Pavlovian Cues

Long distance projections can be crucial to modulating local neural computations (see above). A recent optogenetic study elegantly showed how phase resetting mediates the translation of single neuron activity to behavior in mice (Courtin et al., 2014), and dovetails with the results of Voloh et al. (2015) showing theta resetting in the medial prefrontal cortex is predictive of the activation of downstream areas as well as of actual behavior. In the study by Courtin et al. (2014), theta oscillations were often apparent when the animals exhibited freezing behavior. Moreover, neural spikes were locked to theta peaks, suggesting that when present, theta oscillations drove local circuit modulation that resulted in a fear response (Figure 5Ai). Intriguingly, the theta phase was reset by the presentation of a Pavlovian cue that predicted the aversive stimulus, and this cue induced phase reset predicted the learned fear response (Figure 5Aii). Notably, theta phase resetting was lacking when the conditioned stimulus was presented, but the fear response was slight, suggesting that without theta-phase resetting, the Pavlovian cue-outcome association was not reactivated. Importantly, optogenetically targeting parvalbumin-expressing interneurons (suggested to be crucial for theta-rhythmic activity; e.g., Wulff et al., 2009), led to disinhibition of pyrimdal cells, phase resetting, and a concomitant fear response. Finally, the authors showed that neurons under optogenetic control projected to downstream structures responsible for initiating the fear response (Figure 5Aiii). In other words, fear responsiveness was dependent on activating specific cell types in the mPFC that learned stimulus-outcome associations, and their effectiveness in activating downstream targets was mediated by theta phase resetting (Figure 5Aiv).

#### Spatial Navigation

A discussion of phase resetting is not complete without the historically important work on spatial navigation in rodents. We will briefly touch on work showing the importance of phase resetting to navigational schemes, but note that many excellent, in-depth reviews have been published on the topic (Buzsáki, 2006; Moser et al., 2008; Buzsáki and Moser, 2013; Lisman and Jensen, 2013).

Precise phase resets triggered by contextual cues are implicated to provide critical location information during spatial navigation by hippocampal circuitry. In particular, the hippocampus is known to be important for spatial navigation in rodents and prominently activates when rodents must remember traversed areas, or planning a route (Pastalkova et al., 2008; Pezzulo et al., 2014). Rodents possess an internal map of space encoded in grid cells (Hafting et al., 2005). These cells form a hexagonal grid independent of their immediate location, which allows derivation of the rodents position and direction in relation to the field encoded by grid cells. A prominent model using such directional information during path integration is the oscillatory interference model (Burgess et al., 2007). In this model, grid cells fire when co-activated by multiple speed- and direction- tuned oscillations (velocity controlled oscillations, VCO). The interference pattern generated by VCOs results in the hexagonal firing pattern of grid cells. In other words, grid cells integrate the input of multiple velocity oscillators, and fire when the peaks of individual oscillators coincide. Thus, the exact phase is crucial in this model, as it sets the internal, spatial representation. For example, in a two-oscillator model, if the phase in one oscillator shifts, then the interference pattern will be broken. To prevent such errors, the phase of the oscillator may be reset to some predefined value by salient cues such as landmarks or reward locations. Such a context dependent signal may be provided by place cells, which fire when the rodent enters the preferred field of the place cell. Thus, a phase reset of VCO by place cells directly leads to a conjunctive map of the space and salient features such as rewards or hairpin turns (Burgess et al., 2007; Hasselmo, 2008).

A separate account of grid cell firing depends on attractor dynamics (Stensola et al., 2012; Yoon et al., 2013). However, such dynamics have been suggested to occur in conjunction with oscillatory interference to optimally modulate grid cell membrane potential (Domnisoru et al., 2013).

### Associative Plasticity and Long Term Memory

We now turn to evidence that phase resetting is critical not only for instantaneous processing of behaviorally relevant stimuli, but also for long-term structural changes in synaptic connectivity. A seminal finding in systems neuroscience is the ability to induce changes in synaptic plasticity via theta-rhythmic stimulation in the hippocampus (Bliss and Lomo, 1973), as well as other brain areas (see Caporale and Dan, 2008). The theta-burst stimulation protocol involves inputting tetanic stimuli at a high frequency (~200 Hz), in 4–6 bursts spaced at a theta period, resulting in long-term synaptic modulation. Either long-term potentiation or depression (that is, a strengthening or weakening of synaptic efficacy) can be produced, depending on when the bursts are applied. If bursts are applied during the peak of endogenous theta, then long-term potentiation is induced; conversely, activation at the trough results in long-term depression (Huerta and Lisman, 1995; Hölscher et al., 1997; Hyman et al., 2003). These findings suggest that for proper synaptic modulation, the phase of theta frequency activation must be properly aligned in relation to a stimulus. In fact, delivering tetanic stimulation either at the peak or trough of *endogenous* theta that is aligned (i.e., “reset”) to stimulus onset determines the direction of synaptic plasticity (McCartney et al., 2004).

This protocol may thus effectively induce synaptic changes because there are intrinsic neurophysiological mechanisms active during theta oscillations in the hippocampus that support synaptic modulation; in other words, this protocol may hijack a naturally occurring phenomenon regulating neural plasticity (Buzsáki, 2002; Canolty and Knight, 2010). Such an interpretation is also consistent with various studies reporting that learning of new stimulus associations is accompanied by increased theta rhythmic neuronal coherence (e.g., Tort et al., 2009; Benchenane et al., 2010). We consequently speculate that naturally occurring synaptic plasticity will also be induced if theta oscillations are reset to an endogenous or exogenous stimulus.

Synaptic plasticity modulated via theta-burst protocols have been most extensively characterized in the hippocampus, though there is evidence that other cortical areas can also be modulated in this way (see Caporale and Dan, 2008). For example, in the human, bursts of 50 Hz at theta-periodic spacing can facilitate long-term potentiation when applied in 10 s bursts, but continuous application results in long-term depression (Huang et al., 2005). Faster oscillations may also facilitate LTP vs. LTD induction, as evidenced by the gamma/beta phase sensitivity of synaptic plasticity in the rodent visual cortex (Wespatat et al., 2004), and may be particularly suited to mechanisms of plasticity that depend on critical windows of spike timing (Fell and Axmacher, 2011). These findings suggest that even in humans, and outside the hippocampus, synaptic plasticity depends on precise rhythmic activity. Given the ubiquitous evidence that plasticity is modulated by oscillatory phase, and that the theta-burst protocol is clinically useful (e.g., Li et al., 2014), future interventional electrophysiological studies in humans should take phase into consideration.

## Methodological Considerations in Inferring Function from Phase Reset Analysis

In the previous section, we described functional correlates of phase aligned activation. Inferring such functional consequences requires explicit consideration of various methodological constraints (Aru et al., 2015). Oscillatory responses can be measured at the local level (e.g., single neurons) or at the macroscopic level (electroencephalography, EEG). Neurons and small circuits of neurons with intrinsic pacemaking and resonance properties contribute to macroscopic oscillatory signatures, but individual units may not oscillate themselves but instead fire sparsely (Kilpatrick and Ermentrout, 2011). Weakly coupled oscillators may interact such that the phase of the individual oscillators is advanced or lagged, and a new phase established after enough cycles. On the other hand, if connections between oscillators are strong enough, they can synchronize even in the presence of stochastic input (Buzsáki and Wang, 2012). A strong impulse can force a phase delay or lag even in weakly coupled oscillators, and reset the oscillator to some initial condition. The relationship of an old phase to a newly established one can be quantified in a phase-transition curve (for review, see Canavier, 2015), and analysis can yield rich insights into the mechanisms and conditions underlying such phase transitioning. A full treatment of the theory of coupled oscillators and its precise predictions is outside the scope of this review (see e.g., references in Canavier, 2015).

### Determining Dominant Oscillations

Establishing that a phase reset has occurred first requires documenting that an oscillation is present (Sauseng et al., 2007), using the tools of signal processing. A general procedure for detecting an oscillation requires: (1) filtering the data in some frequency range based on *a priori* knowledge; and (2) transforming the signal such that it is amenable to time-frequency analysis. Popular transformations include the Discrete Fourier transform, the Hilbert transform, and the wavelet transform, though, properly parameterized, they can yield equivalent results (Bruns, 2004). The latter two convert the signal into an analytic (i.e., complex) signal amenable to analysis of instantaneous amplitude and phase, key characteristics of oscillations evolving in time. Whatever transformation is chosen, a key step is calculating the spectral density of the signal, a technique to define the relative contributions of various frequencies to the observed signal in a particular epoch of interest. Such plots usually exhibit a 1/*f*^a^ power law (Buzsáki, 2006; Cohen, 2014). Oscillations that are present in the signal are thus expected to manifest themselves in the spectral plot as “peaks” or bumps deviating from the 1/*f*^a^ structure. On the other hand, broadband increases in power typically reflect asynchronous activity (Miller et al., 2014). Because lower frequencies contribute more power, peaks in higher frequencies may not be apparent visually (Buzsáki, 2006; Cohen, 2014); a common practice is thus to scale the observed spectral density by *f*^a^, in order to detect peaks. Moreover, small power changes in a frequency of interest may be masked due to background activity, particularly when calculated over single trials (Sauseng et al., 2007). In addition to considering the power spectrum, other methods also consider the duration that spectral peaks are evident for (a proxy for the number of oscillation cycles; Caplan et al., 2001), and the auto-correlation of the signal, which can be powerful in measuring the dominant oscillation in the presence of noise (Masimore et al., 2004; Mureşan et al., 2008).

Traditional signal processing techniques assume a stationary signal, and non-stationaries can lead to spurious oscillations and second degree interactions (Aru et al., 2015). The observed signal might have non-oscillatory as well as oscillatory components. Methods such as the Hilbert Huang transform, which can be used on non-stationary signals, can decompose the signal without *a priori* assumptions of relevant “bands” of activity. This allows detection of empirical modes—which can be either oscillatory or non-oscillatory—that significantly contribute to the signal, thus allowing determination if different modes are functionally relevant. Another way is to take advantage of the task design to dissociate the contributions (or lack thereof) of different signals according to behavior or trial type, and in particular, to show that non-stationarities are not functionally relevant (Aru et al., 2015). Regardless of the control analyses performed, a visual inspection of the raw trace is helpful to understand and verify the characteristics of the dominant oscillation evident in the power spectrum.

### Phase Reset Analysis: Controlling for Potential Confounds

A necessary condition for phase resetting is a concentration of phase after a reference time point across trials. Common measures to determine if the phase is consistent over trials include the inter-trial coherence (Makeig et al., 2002), and the phase-preservation index (PPI; Mazaheri and Jensen, 2006). The inter-trial coherence is calculated by taking, for each time point, the modulus of the circular phase average across trials. The PPI is based upon the prediction that in the case of a phase reset, there should be no relationship between the phase before and after the reset event across trials. In other words, the *difference* in angles between two points in time (e.g., before and after a cue onset) is calculated *within* each trial. The PPI is then calculated by, once again, taking the modulus of the circular phase average. If the phase difference is random across trials, phase resetting has likely occurred; whereas if the phase difference is concentrated, a pre-cue phase influences the post-cue phase, implying that no phase reset occurred. In either case, statistical significance can be assessed via permutation testing, or via the Rayleigh’s test for circular uniformity.

Electrophysiological studies measuring the EEG or LFP typically record their activity in relation to a reference electrode. Imagine a scenario where the reference electrode records an oscillation. In this case, the oscillation will also be evident in all other recording electrodes. This could lead to a spurious conclusion of coherence between the recording electrodes. Moreover, a phase reset in the reference electrode may be incorrectly attributed to a phase reset in the recording electrodes. As a first step, proper placement of the reference can mitigate some of the risks (Cohen, 2014). Additionally, each channel can be referenced separately, again mitigating the risks posed by a common reference. For an in-depth discussion and simulation of the common reference problem, refer to Bastos and Schoffelen (2015) in this issue.

A signal recorded by an electrode may have multiple components contributing to the activation waveform. This could include oscillatory activity in multiple distinct bands (as described above), as well as evoked activity. In the EEG literature, there is a long-running debate on whether the evoked potential observed in relation to variable task contexts is mainly a result of oscillatory or evoked activity, and methods have been proposed to differentiate between the two (Makeig et al., 2004; Shah et al., 2004; Sauseng et al., 2007; Ding and Simon, 2013). A first approximation to determining if phases have been reset is by analyzing the power and phase concurrently, averaged across trials; if the power remains constant in response to a stimulus, but the phase becomes more consistent (across trials), then it is more likely that an oscillation has been reset (Sauseng et al., 2007). However, a recent study has shown that the “total power” (power calculated on each trial, then averaged) is statically relatively weaker than phase consistency at detecting changes in stimulus synchronized activity (Ding and Simon, 2013). On the other hand, “evoked power” (waveforms averaged over trials, then power calculated) was nearly as good as phase consistency measures at detecting such activity. In other words, if the power of the trial-averaged trace does not show a stimulus-driven change, and a new phase (consistent over trials) is established that is locked to a stimulus, then this would constitute strong evidence that a phase reset has occurred.

In analyzing oscillatory signatures, considering the phase and power concurrently can provide evidence of a phase reset, but it is important to consider the electrophysiological modality used to make recordings. In the vast majority of studies on oscillations, recorded signals represent spatially synchronized activity (LFP/MEG/EEG). In such a case, there are three potential mechanisms that could lead to an observed phase reset: (1) a single oscillator that is reset by a stimulus; (2) many individual oscillation generators that become spatially synchronized; or (3) the recruitment of new oscillation generators (Ding and Simon, 2013). In all cases, it is possible for power to change, even in the presence of a phase reset; in (1) the amplitude may increase; while in (2) and (3) spatial synchronization necessarily raises the signal-to noise- ratio for a particular band, which will manifest as a concurrent increase in power (Telenczuk et al., 2010). This underscores the point that our knowledge of neurophysiological circuits is limited, which restricts our ability to make strong inferences about the presence or absence of oscillations (Cohen, 2014). High-density neural recordings, in conjunction with cell-type based imaging and recording, can help disentangle the spatial component of phase-reset signals.

In summary, signal processing methods provide powerful analytic tools for assessing the quality of oscillations and their changes. We have covered some considerations and interpretations of common methods in relation to phase resetting of neural activity and stress that these methods must be interpreted in the context of the underlying neurophysiology and type of recording.

## Conclusion

Coordination of oscillations across anatomical and temporal scales is emerging as a fundamental principle in functional connectivity underlying cognition and behavior (Fries, 2005; Buzsáki, 2006; Siegel et al., 2012; Womelsdorf and Everling, 2015). Phase realignment of oscillations is an observable marker of the changing dynamics that underlie such coordination, and is an important step moving away from static snapshots of brain connectivity and towards dynamically evolving circuits (Kopell et al., 2014). Oscillatory phase carries stimulus information, and likely supports coding schemes prevalent across diverse brain systems. These are mediated by local microcircuits, and the information contained therein is propagated across long distances and according to anatomical connectivity profiles. Future studies are needed with a focus on disentangling the anatomical origins of phase aligned activation, microcircuits that can be preferentially targeted to adjust phase, the effect of noise either in the stimulus or in endogenous oscillatory activity, and methods of manipulating neural populations in order to affect computation and behavior. We believe that understanding the phase resetting properties of neural circuits and their emergence during learning processes will be an essential avenue for future research dedicated to identify how large-scale networks are coordinated during goal directed behavior.

## Author Contributions

BV and TW contributed equally to the literature review and writing of this work.

## Conflict of Interest Statement

The authors declare that the research was conducted in the absence of any commercial or financial relationships that could be construed as a potential conflict of interest.
